# The Earth Is Small for “Leviathans”: Long Distance Dispersal of Giant Viruses across Aquatic Environments

**DOI:** 10.1264/jsme2.ME19037

**Published:** 2019-08-03

**Authors:** Yanze Li, Hisashi Endo, Yasuhiro Gotoh, Hiroyasu Watai, Nana Ogawa, Romain Blanc-Mathieu, Takashi Yoshida, Hiroyuki Ogata

**Affiliations:** 1 Institute for Chemical Research, Kyoto University Uji 611–0011 Japan; 2 Department of Bacteriology, Faculty of Medical Sciences, Kyushu University 3–1–1 Maidashi, Higashi-ku, Fukuoka 812–8582 Japan; 3 Graduate School of Agriculture, Kyoto University Kitashirakawa-Oiwake, Sakyo-ku, Kyoto 606–8502 Japan

**Keywords:** ‘Megaviridae’, *Mimiviridae*, DNA polymerase, MEGAPRIMER, richness

## Abstract

Giant viruses of ‘Megaviridae’ have the ability to widely disperse around the globe. We herein examined ‘Megaviridae’ communities in four distinct aquatic environments (coastal and offshore seawater, brackish water, and hot spring freshwater), which are distantly located from each other (between 74 and 1,765 km), using a meta-barcoding method. We identified between 593 and 3,627 OTUs in each sample. Some OTUs were detected in all five samples tested as well as in many of the *Tara* Oceans metagenomes, suggesting the existence of viruses of this family in a wide range of habitats and the ability to circulate on the planet.

‘Megaviridae’, also referred to as the extended *Mimiviridae*, is a rapidly expanding proposed family of nucleocytoplasmic large DNA viruses (NCLDVs) (see [24, 32] and references therein; [7] for an alternative proposal). ‘Megaviridae’ includes giant viruses such as mimiviruses (20), *Cafeteria roenbergensis virus* (10), and *Prymnesium kappa virus* RF01 (17). Early marine metagenomic surveys revealed the existence of viruses of ‘Megaviridae’ in the sea (13, 25, 26). They were later found to be highly abundant (14) and active (5) across oceanic regions, at an abundance estimated to be 10^3^ to 10^5^ genomes mL^−1^ seawater. The taxon richness of this viral group appears to be very large and exceeds that of the whole domain of *Bacteria* (24), suggesting that a diverse array of eukaryotes are potential hosts of these mostly unidentified viruses. Isolated lineages of this viral family are still rare; they infect unicellular algae (12, 17, 27, 31–33) and aquatic heterotrophic protists, such as amoeba infecting mimiviruses (1, 9, 10, 29). Furthermore, a viral group infecting sturgeons, a group of fishes from which caviar is obtained, was recently shown to be related to ‘Megaviridae’ (8). Therefore, viruses in this group appear to have important, but vastly unknown ecological functions in the aquatic environment through the regulation of the populations of their eukaryotic hosts.

Meta-barcoding approaches using degenerate polymerase chain reaction (PCR) primers that amplify specific genes from environmental DNA have been successful in characterizing the community structures of ‘Megaviridae’ in aquatic environments (18, 21, 37). We recently proposed a set of 82 pairs of degenerate PCR primers (*i.e*., MEGAPRIMER) targeting the B family DNA polymerase genes (*polBs*) of ‘Megaviridae’ (22). These primers were designed based on 904 metagenomic ‘Megaviridae’ *polBs* in addition to 17 *polBs* from viruses with sequenced genomes. The MEGAPRIMER approach revealed 5,595 ‘Megaviridae’ non-singleton operational taxonomic units (OTUs) at a nucleotide sequence identity of 97% in a seawater sample taken at Osaka Bay, Japan. However, MEGAPRIMER was only tested for a single seawater sample in our previous study. Therefore, the effectiveness of MEGAPRIMER has not yet been sufficiently demonstrated. In the present study, we used the same primer set to investigate ‘Megaviridae’ community structures in four different aquatic environments.

Between June 2016 and October 2016, samples were collected from four geographically distant locations in Japan to cover a broad range of aquatic environments (Fig. 1 and Table 1). The sampling sites were distantly located from each other (between 74 and 1,765 km). Osaka Bay is a typical eutrophic environment surrounded by densely populated districts and forests with the input of nutrients from rivers. The Japan Sea represents a marginal sea environment that is semi-isolated from the north Pacific. A mangrove site in Ishigaki Island was selected for the potential existence of ‘Megaviridae’ (25). The Miyuki hot spring of Shirahama was also selected to examine the existence of ‘Megaviridae’ in a high temperature environment. Four liters of surface water (from a depth of between 0 and 5 m) was collected at each sampling location. Filtration and DNA extraction were performed as previously described (22). Each primer pair of MEGAPRIMER was used in a separate PCR amplification as previously described (22). In the present study, we did not select amplicons based on visualization in gel electrophoresis as previously reported (22); we mixed all 82 amplicons and an identical barcode was attached to PCR products from the same sample to distinguish sequences from different samples. One sequencing run was performed using a MiSeq platform with MiSeq V3 (2×300 bp) reagent kits (Illumina, San Diego, CA, USA) and with a spike-in of PhiX at 50% to serve as an internal control. Raw reads were processed using the ‘Megaviridae’ Amplicon Processing System (MAPS) as previously described (22). OTUs were formed using CD-HIT-EST (11) with a nucleotide sequence identity of 97%. Rarefaction curves were generated using matplotlib package version 2.0.2 (16). The most abundant 100 OTUs in each sample were selected (423 OTUs after removing redundancy) and used to build a phylogenetic tree based on their translated sequences. The phylogenetic tree was generated using FastTree version 2.1.9 (28) with a default setting (the JTT+CAT model) and visualized using Python ETE3 package version 3.0.0b35 (15). A Unifrac analysis (23) was performed with the scikit-bio package version 0.5.1 of Python and visualized using R. Metagenomic genes from published *Tara* Oceans data (34) downloaded from MGENES (https://www.genome.jp/mgenes/) were initially screened for homologs of the mimivirus PolB sequence with TBLASTN (2), and then analyzed with CD-HIT-EST to identify genes nearly identical to OTUs (nucleotide sequence identity>97%). Raw read data were deposited to DDBJ (accession number DRA008113), and sequence data are also available from our ftp site (ftp://ftp.genome.jp/pub/db/community/MEGAPRIMER_papers).

The resulting high quality ‘Megaviridae’ *polB* fragments were grouped into 3,627, 1,093, 593, and 220 non-singleton OTUs for Osaka Bay Aug., Japan Sea, Ishigaki Island, and Miyuki hot spring, respectively (Table 2). Rarefaction curves indicated that the number of OTUs was close to the plateau under the sequencing depth examined in the present study (Fig. 2). Unweighted Unifrac distances were calculated between ‘Megaviridae’ community structures in five samples by including previously generated data from Osaka Bay (22) and by selecting the 200 most abundant OTUs from each sample. A multidimensional scaling (MDS) analysis showed that the two samples from Osaka Bay were similar to each other. Other samples (Japan Sea, Ishigaki Island, and Miyuki hot spring) were distantly placed from the Osaka Bay samples in the MDS plot (Fig. 3).

We then performed phylogenetic analyses of representative sequences. The tree revealed dozens of diverse clades (clades *ii* to *xii* in Fig. 4) for ‘Mesomimivirinae’ (a proposed subfamily of ‘Megaviridae’), which includes known viruses of unicellular algae. The tree also revealed the detection of sequences belonging to ‘Megamimivirinae’ (another proposed subfamily including mimiviruses, *Cafeteria roenbergensis virus* and klosneuviruses; clade *i* in Fig. 4). However, we did not detect any sample-specific clades, which was unexpected because the hot freshwater spring (Miyuki) appeared to be ecologically distinct and isolated from other sites. In other words, each clade was found to contain OTUs from all or nearly all samples, although there were also OTUs specific to individual samples (as indicated by triangles in the outer ring of Fig. 4). Furthermore, a large proportion (78%; 330 OTUs) of the selected OTUs were found in more than one sample and 17 OTUs (4%) were discovered in all samples (Fig. 5). Regarding 7 out of 17 OTUs, their presence across five samples was supported at an identical read level (*i.e*. identical genotypes; star marks in Fig. 4).

The large number of OTUs shared among the samples tested prompted us to search metagenomic genes from the previous *Tara* Oceans expedition (34), which covered a large part of global oceans, for the non-singleton OTUs identified in the present study. Many of the OTUs were discovered in different oceanic regions, including the opposite side of the earth from Japan, such as the South Atlantic Ocean (Table 3).

Previous studies isolated highly similar giant viruses in different countries. The genome of *Mimivirus shirakomae* (GenBank: AP017645) isolated in Japan (35) was nearly identical (nucleotide sequence identity of ~99.9%) to the genome of the first mimivirus (GenBank: NC_014649) isolated in England (29). The genome of a marseillevirus isolated in Shanghai (GenBank: MG827395) was nearly identical (~98.5%) to the genome of a marseillevirus isolated from Cannes, France (GenBank: KF261120). These findings suggest the long distance dispersal of giant viruses across continents and oceans through unidentified mechanisms, possibly via microscale droplets (30, 36), wind (3, 19), or oceanic current systems (4), as suggested for other smaller viruses. In the present study, we revealed the existence of *polB* OTUs that may be observed in largely distinct aquatic ecosystems, which span seawater, a mangrove site (brackish water), and freshwater hot spring. Furthermore, some OTUs were found in different oceans. Therefore, the dispersal of ‘Megaviridae’ occurs across distant geographical locations on a global scale. The present results also imply a relatively wide habitat and niche for at least some of the viruses belonging to ‘Megaviridae’.

It is notable that we detected 220 ‘Megaviridae’ OTUs from Miyuki hot spring, at which the water temperature was 69.4°C. This result suggests the existence of diverse ‘Megaviridae’ in a high temperature environment. Giant viruses have not yet been isolated from an environment as hot as Miyuki spring, except for medusavirus recently isolated from a cooler, but still warm environment (43.4°C freshwater) (38). A previous study reported the genome sequences of ‘Megaviridae’ assembled from metagenomic samples from a hot spring site, Yellowstone Lake (39). However, the genomes were co-assembled from different metagenomic data derived from samples collected at different locations with varying temperatures between 10 and 96°C. Therefore, it currently remains unclear whether ‘Megaviridae’ exist in a hot or warm environment. Another related study on the same metagenomes revealed the existence of virophages, which are parasites of ‘Megaviridae’ viruses, in a high temperature ecosystem (40). Zhou *et al*. assembled seven virophage genomes from 42 samples from Yellowstone Lake, and all of the virophage genomes were detected in vent water metagenomic samples (between 40 and 68°C). In the literature, the upper temperature limit for a eukaryotic cell to reproduce has been described as 65°C (6). Therefore, there may be no actively replicating eukaryotic cells in hot water at 69.4°C. In the Miyuki hot spring sampling site, water runs into a drain open to the surrounding environment, including the atmosphere. Therefore, it currently remains unclear whether ‘Megaviridae’ actively infect eukaryotic hosts in a hot environment. The quantitativity of the MEGAPRIMER approach needs to be investigated as previously pointed out (22); however, the two Osaka Bay samples (collected in different years, but within similar periods) being placed closely to each other in the MDS plot (Fig. 3) corroborate the effectiveness of the MEGAPRIMER approach for comparisons of ‘Megaviridae’ communities across environments.

## Figures and Tables

**Fig. 1:**
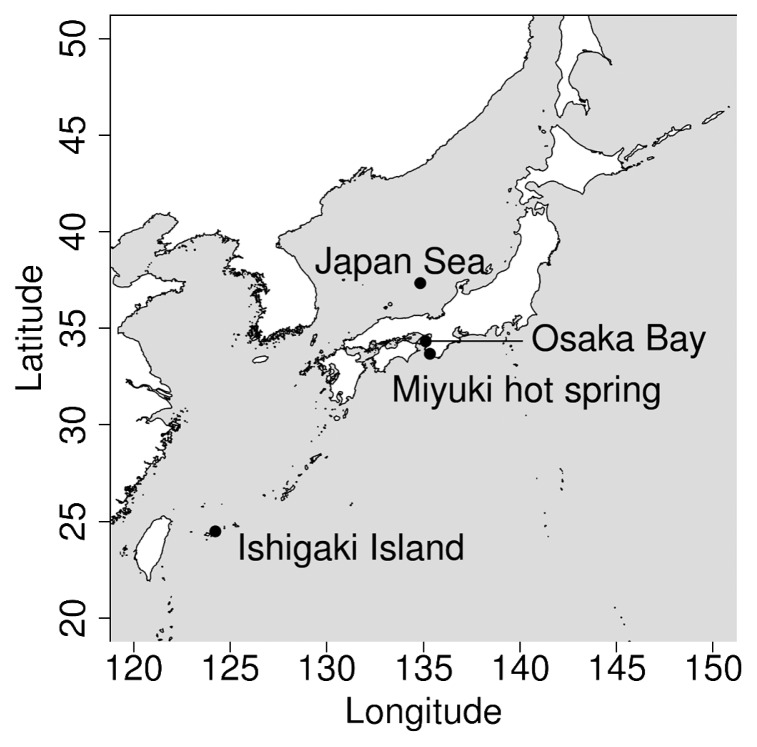
Locations of four sampling sites.

**Fig. 2:**
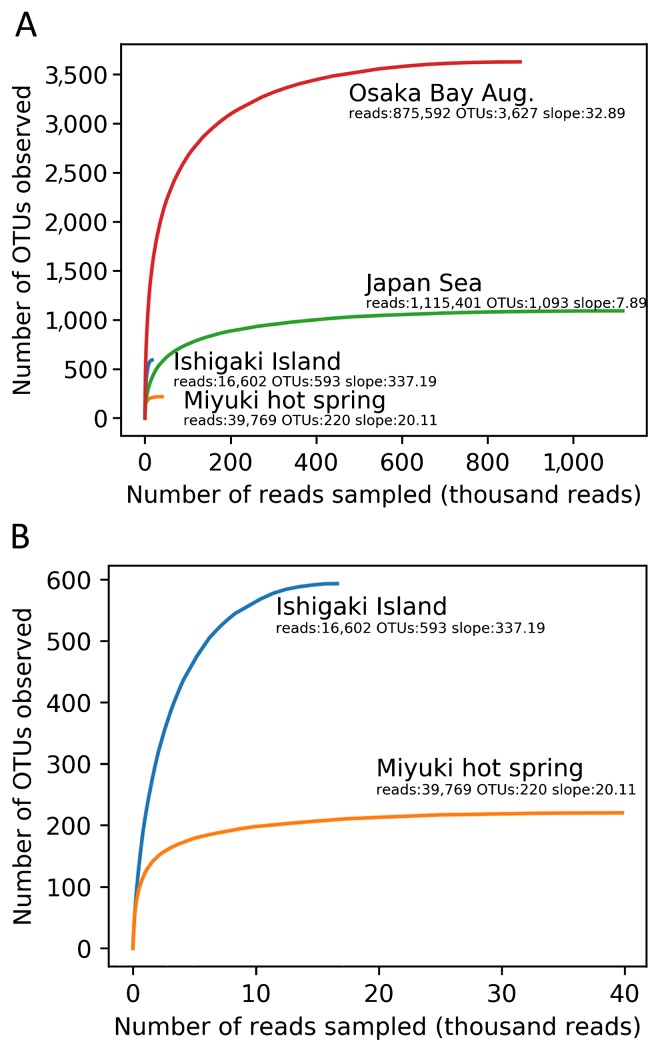
Rarefaction curves for Megaviridae *polB* OTUs with a 97% DNA sequence identity cut-off for four samples. (A) All four samples. (B) A magnified view of Ishigaki Island and Miyuki hot spring samples. Proximity to saturation is indicated by weak slopes at the end of each rarefaction curve. For example, an increase in 32.89 OTUs per resampling of one million reads is noted as a slope of 32.89/1M.

**Fig. 3:**
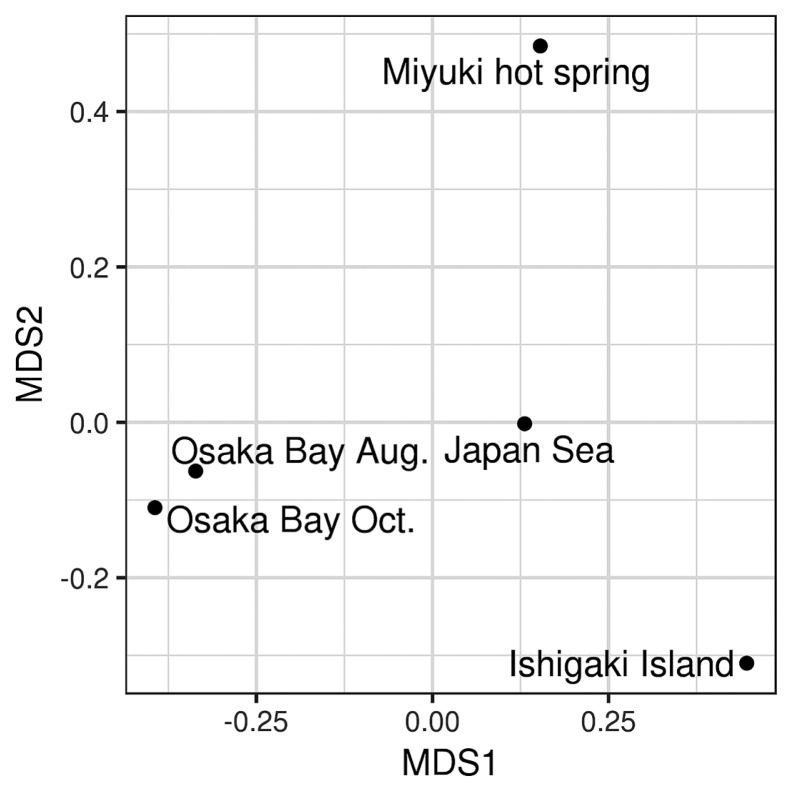
MDS analysis of pairwise unweighted Unifrac distances between five samples. The stress value was 2.5×10^−14^.

**Fig. 4:**
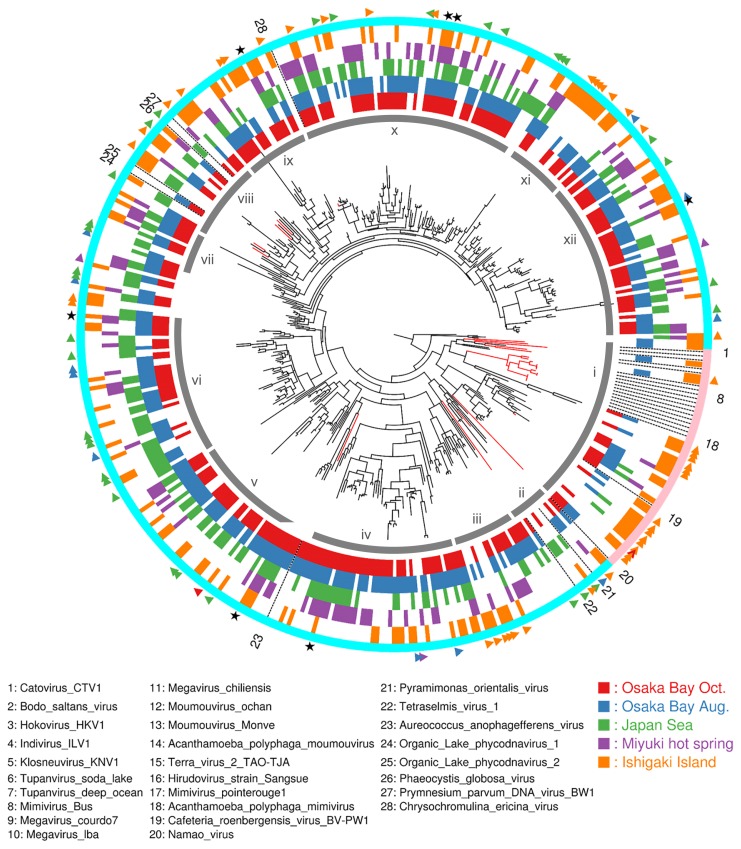
Maximum-likelihood phylogenetic tree of ‘Megaviridae’ PolB meta-barcodes with additionally known ‘Megaviridae’ sequences. The tree is rooted with nine *Phycodnaviridae* sequences, which are not shown in this figure. Leaves are either meta-barcodes (black) or reference ‘Megaviridae’ PolBs (red). Triangles in the most outer ring indicate OTUs specific to a single sample, and stars indicate OTUs containing genotypes shared among all samples. The next ring from outside indicates putative ‘Megamimivirinae’ (pink) or putative ‘Mesomimivirinae’ (light blue). The next five rings indicate the presence/absence of OTUs in the respective samples. The gray lines (labeled with Roman numerals from *i* to *xii*) inside the presence-absence rings indicate major clades of OTUs.

**Fig. 5:**
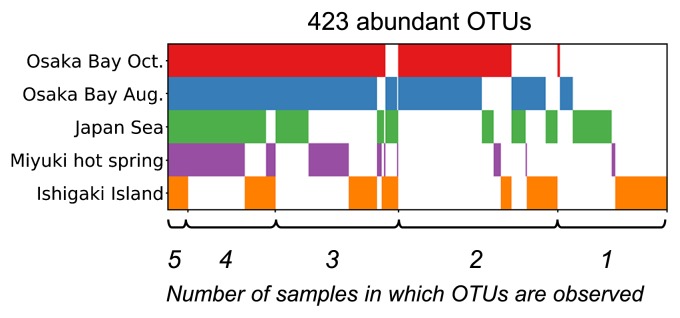
Overlap of the presence of abundant OTUs across five samples. The abundant OTUs shown in Fig. 4 (selection of 100 of the most abundant OTUs from each sample; 423 OTUs in total) correspond to columns of the matrix, while the five samples correspond to rows. OTUs were sorted from left to right according to the number of samples in which they were observed. The presence of an OTU in a sample is indicated by colors: red (Osaka Bay Oct.), blue (Osaka Bay Aug.), green (Japan Sea), purple (Miyuki hot spring), or orange (Ishigaki Island). OTUs at the leftmost side are present in all five samples, while OTUs on the rightmost side are present only in the Ishigaki Island sample. A large proportion (78%) of OTUs were found in more than one sample.

**Table 1:** Locations and sampling dates of samples used in the present study

Sample	Longitude	Latitude	Depth (m)	Temperature (°C)	Salinity	Date	Reference
Osaka Bay Oct.	N 34°19′28″	E 135°7′15″	5	22.01	33.10	October 30, 2015	(22)
Osaka Bay Aug.	N 34°19′28″	E 135°7′15″	5	25.62	32.51	August 22, 2016	This study
Japan Sea	N 37°20′06″	E 134°49′85″	0	25.5	32.03	July 25, 2016	This study
Ishigaki Island	N 24°19′19″	E 124°03′23″	0	29.5	NA*	October 13, 2016	This study
Miyuki hot spring	N 33°40′35″	E 135°20′18″	0	69.4	0.8	June 21 2016	This study

* The salinity meter broke down at this sampling site.

**Table 2:** Number of operational taxonomic units (OTUs) for each sample.

Sample	Total number of OTUs	Number of singleton OTUs	Number of non-singleton OTUs	Number of sequences included in non-singleton OTUs
Osaka Bay Aug.	5,653	2,026	3,627	875,592
Japan Sea	2,206	1,113	1,093	1,115,401
Ishigaki Island	881	288	593	16,602
Miyuki Hot spring	304	84	220	39,769

**Table 3:** Number of OTUs identified in *Tara* Oceans data.

Sample	Oceanic region*							

	MS	RS	IO	SAO	SO	SPO	NPO	NAO
Osaka Bay Oct.	35	29	146	119	1	224	73	53
Osaka Bay Aug.	37	9	96	79	0	159	58	28
Japan Sea	17	2	46	58	0	63	27	15
Ishigaki Island	13	1	28	21	0	26	12	5
Miyuki hot spring	6	2	18	12	0	19	9	11

* Abbreviations: MS, Mediterranean Sea; RS, Red Sea; IO, Indian Ocean; SAO, South Atlantic Ocean; SO, Southern Ocean; SPO, South Pacific Ocean; NPO, North Pacific Ocean; NAO, North Atlantic Ocean.
